# AI As the New Age Estimator: Pioneering Customized Facial Surgery Outcomes

**DOI:** 10.1093/asjof/ojae007.087

**Published:** 2024-04-12

**Authors:** Khaled Alameddine, Jess Rames, Karim Bakri, Samir Mardini

**Affiliations:** Mayo Clinic, Rochester, MN; Mayo Clinic, Rochester, MN; Mayo Clinic, Rochester, MN; Mayo Clinic, Rochester, MN

## Abstract

**Goals/Purpose:**

The imperative for precision in aesthetic surgery necessitates a robust framework for evaluating the impact of facial interventions on perceived age. Our study introduces a cutting-edge AI model aimed at discerning an individual's perceived age from facial characteristics. This tool is designed to augment the assessment of various plastic surgery procedures, facilitating the tailoring of interventions to each patient's unique facial aging pattern.

**Methods/Technique:**

We harnessed a deep convolutional neural network (DCNN), pre-trained on the extensive ImageNet dataset, and further refined using 523,051 pre-annotated facial images from the IMBD-WIKI database, normalized as per the Mathias et al. face detection paradigm. Faces were processed into a 299x299 pixel matrix, maintaining a 40% margin around the face for uniformity. The Xception architecture was employed for its advanced feature extraction capabilities. The model was refined and tested against a diverse set of 100 patient faces from the Mayo Clinic's database, categorized by demographic and procedural data. The AI model employed regression analysis and softmax probability for precise age estimation.

**Results/Complications:**

The AI model exhibited a remarkable accuracy rate of 92.5% in age estimation for pre procedural patients, with a standard deviation of 3.2 years. It significantly outperformed traditional methods in identifying fine-grained age-related features. The AI model discerned an average perceived age reduction of 3.5 years across all patients post-procedure, with a notable variance among different types of surgeries. Certain procedures, such as rhytidectomy and blepharoplasty, showed a more pronounced age-reduction effect.

**Conclusion:**

The AI model presents an accurate and objective method for quantifying perceived age, serving as a significant benchmark in facial aesthetic evaluation. By illustrating measurable age reduction following various procedures, with some surgeries yielding more substantial changes in perceived age, the model stands as a testament to the effectiveness of plastic surgery interventions. The precision of our model in predicting age pre- and post-procedure underscores its potential to assist surgeons in custom-tailoring surgeries to individual aging patterns. This innovation is poised to refine the decision-making process in aesthetic surgery, ensuring treatments are aligned with the desired outcomes for rejuvenation and patient-specific needs, ultimately advancing the frontier of personalized plastic surgery.

